# Bone Demineralization in Postmenopausal Women: Role of Anamnestic Risk Factors

**DOI:** 10.1155/2012/837187

**Published:** 2012-07-26

**Authors:** Sandro La Vignera, Rosita A. Condorelli, Enzo Vicari, Chiara Nicoletti, Aldo E. Calogero

**Affiliations:** Section of Endocrinology, Andrology and Internal Medicine, Department of Medical and Pediatric Sciences, University of Catania, Policlinico, 95123 Catania, Italy

## Abstract

This study evaluated the effects of LT4 administration on the bone mineral density (BMD) in physiological postmenopausal women after two years of continuative treatment. 110 postmenopausal women with nodular goiter aged between 50 and 55 years were examined before and after 2 years of therapy with a fixed dose of LT4 (1.6 mcg/kg/die) for the treatment of nodular thyroid disease. The results showed that the patients on treatment with LT4 have a slight, but significant reduction of the BMD after 2 years of treatment, associated with increased serum levels of alkaline phosphatase and urinary excretion of hydroxyproline, confirming our data conducted on the same group after one year of therapy. Comparison between patients receiving LT4 (group A) or not (group B) showed that group A patients had significantly lower BMD. We demonstrated the statistically significant influence of the following risk factors on BMD: (1) body mass index <19 kg/m^2^; (2) the onset of menarche after the age of 15 years; (3) positive history for period of amenorrhoea; (4) nulliparity.

## 1. Introduction

Bioavailable 17*β*-estradiol correlates with the bone mineral density (BMD). On the contrary, an estrogenic lack determines a condition characterized by the prevalence of bone reabsorption phase over the bone formation phase. Osteoporosis in postmenopause recognizes also low serum 1,25(OH)_2_D levels and a reduced intestinal transport of calcium, always due to the estrogenic lack. The reabsorption markers increase up to two times the values seen in premenopause, while values of bone formation markers increase only by 50% [1]. The biochemical indicators of increased osteoblastic activity are represented by the serum levels of osteocalcin and of alkaline phosphatase, while the urinary levels of hydroxyproline are expression of an increased osteoclastic activity. In presence of an accelerated bone replacement, osteocalcin and alkaline phosphatase serum levels and urinary hydroxyproline excretion are increased. The diagnosis of osteopenia and/or osteoporosis is conventionally made by measuring bone densitometry. There is a wide debate concerning the effects of the treatment with L-thyroxin (LT4) on BMD. Thyroid hormones certainly determine an increase of both osteoblastic and osteoclastic activities over both cortical and trabecular bones [2, 3]. However, the data about the real impact that LT4 therapy exerts on BMD of women subjected to prolonged periods of treatment appear contrasting. In particular, an approach built on the preventive determination of the anamnestic profile considered at risk for bone demineralization in women undergoing LT4 use is lacking. Hyperthyroidism has been associated with bone demineralization [3–7]. Studies in vitro suggest that thyroid hormones increase more the re-absorption than bone formation, hence determining a loss of bone mass [8, 9]. It has also been reported how thyrotoxicosis increases the fracture risk in post-menopause women and in patients with low bone mass peak. Fractures are more frequent in those parts of the skeleton in which predominates the cortical bone, such as the hip and the distal part of the arm [10]. A recent study has also shown that endogenous subclinical hyperthyroidism might be considered an additional risk factor for osteoporosis in postmenopausal women, especially for cortical bone, whereas exogenous subclinical hyperthyroidism has no effect on BMD [11]. Post-menopausal women with nodular thyroid disease often undergo continuative and prolonged treatment with LT4 administered at suppressive dosages. Nodular thyroid disease and osteoporosis share common factors: (a) both are present with an elevated frequency in the general population; (b) they are more prevalent in the female sex; (c) the incidence increases with age. Since increased levels of thyroid hormones may contribute to bone demineralization.

The aim of this study was to evaluate the effects of treatment with a fixed dose of LT4 on BMD in physiological post-menopausal women with normofunctioning nodular thyroid disease, compared to women, the same average age, who did not receive LT4.

## 2. Patients and Methods

### 2.1. Patient Selection

To examine the effects of the treatment with LT4 on the BMD, 110 (range 50–55 years, average age 53), natural post-menopausal patients with normo-functioning uninodular or multinodular benign thyroid disease were enrolled, after informed consent, if they:

- had last regular menstruation less than 5 years before;
- had a normal T-score (≥1 SD);
- did not have secondary causes of bone demineralization (genetic, endocrine-metabolic, osteoarticular, hematologic, neoplastic, gastrointestinal, drug administration, and/or prolonged immobilization);
- did not receive LT4 treatment in the previous two years;
- did not have a history positive for vertebral fracture.

Following preliminary evaluation, all patients were prescribed LT4 at the fixed dose of 1.6 *μ*g/kg/die.

**Exclusion Criteria**

Surgical menopause, hormone replacement therapy in the last two years and autoimmune thyroid disorders.

### 2.2. Control Group

Fifty women of the same average age (range 50–55 yrs, average age 53.5), having their last menstruation less than 5 years before (last referred menstruation), with normal T-score (≥1 SD) and not receiving LT4.

None of the patients assumed any drugs. 

### 2.3. Instrumental and Laboratory Evaluation

All patients underwent evaluation of BMD by dual X-ray absorptiometry of the lumbar vertebrae (Bone Densitomiters Gammadensit X-ray, l'ACN Scientific Laboratories; Cerro Maggiore (MI) Italy) before LT4 treatment was begun (T0) and 2 years after treatment (group A) and/or followup (group B) (T2). The serum levels of TSH (ECLIA, Roche Diagnostics, Monza (MI), Italy), FT4, FT3 (ECLIA, Roche Diagnostics, Monza (MI), Italy), AbTG and AbTPO (ECLIA, Roche Diagnostics, Monza (MI), Italy), were evaluated at T0 and at T2. All the patients enrolled underwent measurements of calcium serum levels (colorimetric method; Roche Diagnostics, Monza (MI), Italy), 24 h urinary calcium concentration (colorimetric method, Roche Diagnostics, Monza (MI), Italy), serum alkaline phosphatase (AP) bone isoenzyme levels (enzymatic method; Metra TM BAP EIA) and 24 h urinary hydroxyproline concentration (colorimetric method; LTA; Bussero (MI), Italy) at T0 and T2.

### 2.4. Statistical Analysis

The results are reported as mean ± SEM throughout the study. According to LT4 therapy, patients were divided into 2 groups: a group which received LT4 therapy (group A) and group which did not receive any treatment (group B). The statistical analysis was performed using the Student *t*-test for paired or unpaired data, as suitable. The odd's ratios of the risk factors in relationship to bone mineralization loss were determined. The statistical significance was accepted when the value of *P* resulted lower than 0.05.

## 3. Results

The patients enrolled had a mean age of 53.4 years (range 50–55 yrs). As expected, the treatment with LT4 caused a significant reduction of TSH serum levels (*P *< 0.0001) at T2. At T0, the group A patients had a mean T-score of −0.22 ± 0.07 which decreased significantly to −1.02 ± 0.10 at T2 (*P *< 0.001). The total calcium concentration in serum and 24 h urine after treatment with LT4 did not change significantly in comparison to the pretreatment values (Tables 1 and 2). The serum levels of AP and the amount of hydroxyproline excreted with the 24 h urine increased significantly (*P *< 0.0001) during treatment with LT4 (Tables 1 and 2). The impact of some risk factors for osteoporosis was evaluated on the BMD of the patients treated with LT4. The results of this analysis are shown in Table 3. A positive history for osteoporosis, smoking habit, and length of menopause did not give a significant odd ratio. In contrast, the following risk factors turned out to influence in a statistically significant manner the BMD: BMI < 19 kg/m^2^, the onset of menarche after the age of 15 years, a positive history for periods of prolonged amenorrhea, nulliparity.

## 4. Discussion

The results of this study showed that patients with nodular thyroid disease treated with a fixed dose of LT4 have a slight, but highly significant reduction of the BMD after two years of treatment. Forty-one out of 110 women (37.3%) had osteopenia at T2 (tra −1 e T-score −2.5 SD); in the control group osteopenia at T2 occurred in 6% of patients. The loss of BMD appeared mainly related to the following factors: (1) BMI < 19 kg/m^2^; (2) menarche onset after the 15th year of age; (3) a clinical history positive for periods of secondary amenorrhea; (4) nulliparity.

In parallel with the modifications of the BMD after two years of LT4 treatment, it was also observed an increase in 24 h urinary hydroxyproline concentration, suggestive of increased osteoclastic activity, and increased serum AP levels, an index of increased osteoblastic activity. Altogether therefore these data suggest that LT4 treatment increases bone metabolic turnover, with prevalence of the re-absorption phase. The results of this study, therefore, favor the hypothesis that the treatment with suppressive dosages of LT4, frequent in the clinical practice of women in menopause with nodular thyroid disease, is a risk factor for the progression of bone demineralization. In particular, women with the following predisposing risk factors: thinness, delayed menarche, periods of secondary amenorrhea during the reproductive age, nulliparity, are likely to undergo a more profound negative impact of LT4 treatment on BMD.

Contrasting results have been published about the role played by LT4 treatment on the BMD. The discrepancies between the various studies are determined by methodological bias, such as (a) progressive reduction of the LT4 dosage prescribed to women which by itself may justify a reduced risk for bone loss; (b) inclusion of patients with a clinical history positive for hypo- or hyper-thyroidism which has been reported to impair BMD [25]; (c) differences in the duration of the treatment; (d) heterogeneous methods and site of BMD evaluation; (e) lack of data collection relative to the role of other possible interfering factors; (f) inclusion of pre-menopausal women; (g) unmatched controls (for body weight, age at menarche and at menopause, calcium dietary intake, smoking habits, alcohol intake, exercise, etc.). A meta-analysis study, published in 1996, reviewed 41 controlled cross-sectional studies, including about 1250 patients, concerning the impact of thyroid hormone therapy on BMD. Suppressive LT4 therapy was associated with significant bone loss in postmenopausal women (but not in premenopausal women), whereas, conversely, replacement therapy was associated with bone loss in premenopausal women (spine and hip), but not in postmenopausal women. The detrimental effect of thyroid hormones appeared more marked on cortical bone than on trabecular bone [26]. We will briefly review the major studies appeared in literature afterwards which can be divided into those showing a detrimental effect of LT4 on BMD and those showing no such an effect [12–24]. A summary of the main features of these studies is reported in Table 4.

A careful selection of a group of post-menopausal women with thyroid nodular disease under treatment with a fixed doses of LT4 allowed us to show that this treatment causes a slight but significant reduction of BMD. In addition, BMD reduction was associated with the following osteoporosis risk factors: BMI < 19 kg/m^2^, the onset of menarche after the age of 15 years, history positive for period of amenorrhea, and nulliparity. In consideration that LT4 treatment is effective in a low percentage of patients with benign thyroid nodules, estimated to be 10–20% [27], a careful benefit/risk evaluation has to be taken into account before LT4 treatment is prescribed, particularly when other risk factors for bone demineralization are present. The present study confirms comments from our previous article, conducted on 99 post-menopausal women [28], all of them receiving LT4, prospectively evaluated after one-year treatment. In addition, to previous data, the present study consider an homogeneous control group, for average age and clinical features, including only physiological menopause limited to 5-year history (last menstruation). It also increase to two-year observation length of women receiving therapy, allowing a better understanding of the phenomenon. Moreover, this study excludes conditioning anamnestic factors such as: surgical menopause, HRT, andautoimmune thyroid disorders. 

## Figures and Tables

**Table 1:** Bone mineral density (BMD), TSH and markers of osteoblastic and osteoclastic activities before (T0) and two years (T2) after treatment with L-thyroxin in 110 postmenopausal patients with nodular thyroid disease.

	T0	T1
BMD (T-score)	−0.22 ± 0.07	−1.02 ± 0.10*
TSH (*μ*IU/mL)	1.3 ± 0.1	0.8 ± 0.07*
Serum calcium concentration (mg/dL)	8.9 ± 0.02	8.8 ± 0.03
Urinary calcium excretion (mg/24 h)	183.2 ± 3.1	190.4 ± 3.3
Alkaline phosphatase (UI/L)	169.7 ± 2.5	188.6 ± 2.3*
Urinary hydroxyproline excretion (mg/m^2^/24 h)	12.8 ± 0.6	15.7 ± 0.4*

*P < 0.001 versus T0.

Normal values: TSH: 0.49–4.67 *μ*U/L; serum calcium concentration: 8.2–10.5 mg/dL; urinary calcium excretion: 50–250 mg/24 h; alkaline phosphatase: 98–279 UI/L; urinary hydroxyproline excretion (22–65 yrs): 6–22 mg/m^2^/24 h.

**Table 2:** Bone mineral density (BMD), TSH and markers of osteoblastic and osteoclastic activities before (T0) and two years (T2) after followup in 50 postmenopausal patients did not receive any therapy (control group).

	T0	T1
BMD (T-score)	−0.27 ± 0.02	−0.30 ± 0.07
TSH (*μ*IU/mL)	1.4 ± 0.2	1.5 ± 0.04
Serum calcium concentration (mg/dL)	9.1 ± 0.06	9.2.8 ± 0.01
Urinary calcium excretion (mg/24h)	144.2 ± 2.1	146.4 ± 2.2
Alkaline phosphatase (UI/L)	160.7 ± 1.5	160.9 ± 1.2
Urinary hydroxyproline excretion (mg/m^2^/24 h)	13.1 ± 0.6	13.0 ± 0.1

*P < 0.001 versus T0.

Normal values: TSH: 0.49–4.67 *μ*U/L; serum calcium concentration: 8.2–10.5 mg/dL; urinary calcium excretion: 50–250 mg/24 h; alkaline phosphatase: 98–279 UI/L; urinary hydroxyproline excretion (22–65 yrs): 6–22 mg/m^2^/24 h.

**Table 3:** Odd's ratio of each risk factor.

Risk factors	*n*	T score <–1	T score > −1 < −2.5	Odd's ratio	95% confidence interval	Chi square (*P* value)
Familiarity +	48	26	16	1.0	0.45–2.4	NS
Familiarity −	62	36	21			
BMI < 19 kg/m^2^	41	11	24	8.6	3.55–21.87	<0.0005
BMI ≥ 19 kg/m^2^	69	51	13			
Menarche > 15 years	50	20	24	3.9	1.64–9.16	<0.0005
Menarche < 15 years	60	42	13			
Amenorrhoea +	52	15	26	7.4	2.97–18.47	<0.0005
Amenorrhoea −	58	47	11			
Nulliparity +	48	12	25	8.7	3.41–22.07	<0.0005
Nulliparity −	62	50	12			
Smoking +	40	17	16	2.0	0.86–4.75	NS
Smoking −	70	45	21			

NS: not statistically significant.

**Table 4:** Summary of the principal characteristics of the main studies exploring the effects of the treatment with L-thyroxin on the bone mineral density (BMD).

Authors	Thyroid disease	Number of patients	Type of treatment	Length of treatment (years)	Menopausal status	Effect on BMD
Affinito et al., 1996 [12]	Hypothyroidism	54	Suppressive	Various	After	Decreased
Baldini et al., 2002 [13]	Nontoxic goiter	43	Suppressive	≥2	Before and after	Unchanged
Chen et al., 2004 [14]	Cancer (thyroidectomy)	69	Suppressive	7.3 ± 3	44 before 25 after	Decreased
	Nodular goiter	32	None	NA		None
Foldes et al., 1993 [15]	Subclinical hyperthyroidism	37	None	NA	Before and after	None
	Toxic adenoma	22	None	NA		Decreased in postmenopausal
Gorres et al., 1996 [16]	Cancer (thyroidectomy)	65	Suppressive		15 before 32 after 18 men	Unchanged
Hadji et al., 2000 [17]	Nontoxic goiter and hypothyroidism	156	Substitutive	>5	Before and after	Slightly decreased
Heijckmann et al., 2005 [18]	Cancer	59	Suppressive	>6		Unchanged
Kung and Yeung, 1996 [19]	Cancer (thyroidectomy)	46	Suppressive	2	After	Decreased
Larijani et al., 2004 [20]	Thyroid nodules	41	Substitutive	>1	Before	Unchanged
Mikosch et al., 2001 [21]	Cancer	98	Suppressive			Unchanged
Nuzzo et al., 1998 [22]	Nontoxic goiter	40	Suppressive	1.5–14	Before	Unchanged
Sijanovic and Karner, 2001 [23]	Cancer (thyroidectomy)	19	Suppressive	9	Before	Decreased
Toivonen et al., 1998 [24]	Cancer (thyroidectomy)	29	Suppressive	9–11	25 women 4 men	Decreased

NA: not applicable.

## References

[1] Garnero P, Hausherr E, Chapuy MC (1996). Markers of bone resorption predict hip fracture in elderly women: the EPIDOS prospective study. *Journal of Bone and Mineral Research*.

[2] Mosekilde L, Melsen F, Bagger JP, Myhere-Jensen O, Sorensen NS (1977). Bone changes in hyperthyroidism: interrelationships between bone morphometry. Thyroid function and calcium phosphorus metabolism. *Acta Endocrinologica*.

[3] Foldes J, Lakatos P (1996). Thyroid and osteoporosis. *European Journal of Nuclear Medicine and Molecular Imaging*.

[4] Mosekilde L, Eriksen EF, Charles P (1990). Effects of thyroid hormones on bone and mineral metabolism. *Endocrinology and Metabolism Clinics of North America*.

[5] Harvey RD, McHardy KC, Reid IW (1991). Measurement of bone collagen degradation in hyperthyroidism and during thyroxine replacement therapy using pyridinium cross-links as specific urinary markers. *Journal of Clinical Endocrinology and Metabolism*.

[6] Greenspan SL, Greenspan FS (1999). The effect of thyroid hormone on skeletal integrity. *Annals of Internal Medicine*.

[7] Vestergaard P, Mosekilde L (2003). Hyperthyroidism, bone mineral, fracture risk—a meta-analysis. *Thyroid*.

[8] Wartofski L (1988). Osteoporosis: a growing concern for the thyroidologist. *Thyroid Today*.

[9] Baran DT, Braverman LE (1991). Thyroid hormones and bone mass. *Journal of Clinical Endocrinology and Metabolism*.

[10] Zatońska K, Bolanowski M (2000). The influence of thyrotoxicosis and thyroxine therapy on the risk of osteoporosis. *Polski Merkuriusz Lekarski*.

[11] Belaya ZE, Melnichenko GA, Rozhinskaya LY (2007). Subclinical hyperthyroidism of variable etiology and its influence on bone in postmenopausal women. *Hormones*.

[12] Affinito P, Sorrentino C, Farace MJ (1996). Effects of thyroxine therapy on bone metabolism in postmenopausal women with hypothyroidism. *Acta Obstetricia et Gynecologica Scandinavica*.

[13] Baldini M, Gallazzi M, Orsatti A, Fossati S, Leonardi P, Cantalamessa L (2002). Treatment of benign nodular goitre with mildly suppressive doses of L-thyroxine: effects on bone mineral density and on nodule size. *Journal of Internal Medicine*.

[14] Chen CH, Chen JF, Yang BY (2004). Bone mineral density in women receiving thyroxine suppressive therapy for differentiated thyroid carcinoma. *Journal of the Formosan Medical Association*.

[15] Foldes J, Tarjan G, Szathmari M, Varga F, Krasznai I, Horvath C (1993). Bone mineral density in patients with endogenous subclinical hyperthyroidism: is this thyroid status a risk factor for osteoporosis?. *Clinical Endocrinology*.

[16] Gorres G, Kaim A, Otte A, Gotze M, Muller-Brand J (1996). Bone mineral density in patients receiving suppressive doses of thyroxine for differentiated thyroid carcinoma. *European Journal of Nuclear Medicine*.

[17] Hadji P, Hars O, Sturm G, Bauer T, Emons G, Schulz KD (2000). The effect of long-term, non-suppressive levothyroxine treatment on quantitative ultrasonometry of bone in women. *European Journal of Endocrinology*.

[18] Heijckmann AC, Huijberts MSP, Geusens P, de Vries J, Menheere PP, Wolffenbuttel BH (2005). Hip bone mineral density, bone turnover and risk of fracture in patients on long-term suppressive L-thyroxine therapy for differentiated thyroid carcinoma. *European Journal of Endocrinology*.

[19] Kung AW, Yeung SS (1996). Prevention of bone loss induced by thyroxine suppressive therapy in postmenopausal women: the effect of calcium and calcitonin. *Journal of Clinical Endocrinology and Metabolism*.

[20] Larijani B, Gharibdoost F, Pajouhi M (2004). Effects of levothyroxine suppressive therapy on bone mineral density in premenopausal women. *Journal of Clinical Pharmacy and Therapeutics*.

[21] Mikosch P, Jauk B, Gallowitsch HJ, Pipam W, Kresnik E, Lind P (2001). Suppressive levothyroxine therapy has no significant influence on bone degradation in women with thyroid carcinoma: a comparison with other disorders affecting bone metabolism. *Thyroid*.

[22] Nuzzo V, Lupoli G, del Puente A (1998). Bone mineral density in premenopausal women receiving levothyroxine suppressive therapy. *Gynecological Endocrinology*.

[23] Sijanovic S, Karner I (2001). Bone loss in premenopausal women on long-term suppressive therapy with thyroid hormone. *Medscape Women's Health*.

[24] Toivonen J, Tähtelä R, Laitinen K, Risteli J, Välimaki MJ (1998). Markers of bone turnover in patients with differentiated thyroid cancer with and following withdrawal of thyroxine suppressive therapy. *European Journal of Endocrinology*.

[25] Jodar E, Munoz-Torres M, Escobar-Jimenez F, Quesada-Charneco M, Luna Del Castillo JD (1997). Bone loss in hyperthyroid patients and in former hyperthyroid patients controlled on medical therapy: influence of aetiology and menopause. *Clinical Endocrinology*.

[26] Uzzan B, Campos J, Cucherat M, Nony P, Boissel JP, Perret GY (1996). Effects on bone mass of long term treatment with thyroid hormones: a meta-analysis. *Journal of Clinical Endocrinology and Metabolism*.

[27] Gharib H, Mazzaferri EL (1998). Thyroxine suppressive therapy in patients with nodular thyroid disease. *Annals of Internal Medicine*.

[28] La Vignera S, Vicari E, Tumino S (2008). L-thyroxin treatment and postmenopausal osteoporosis: relevance of the risk profile present in clinical history. *Minerva Ginecologica*.

